# Effect of hyperlipidemia on the incidence of cardio-cerebrovascular events in patients with type 2 diabetes

**DOI:** 10.1186/s12944-018-0676-x

**Published:** 2018-05-08

**Authors:** Dabei Fan, Li Li, Zhizhen Li, Ying Zhang, Xiaojun Ma, Lina Wu, Guijun Qin

**Affiliations:** 1 Division of Endocrinology Department of Internal Medicine, The First Affiliated Hospital of Zhengzhou University, Zhengzhou, Henan China; 2 Ophthalmologic Center, The First Affiliated Hospital of Zhengzhou University, Zhengzhou, Henan China

**Keywords:** Hyperlipidemia, Type 2 diabetes, Cardio-cerebrovascular diseases, Hypertension

## Abstract

**Background:**

This study was to explore the effect of hyperlipidemia on the incidence of cardio-cerebrovascular diseases in patients with type 2 diabetes.

**Methods:**

Three hundred ninety five patients with type 2 diabetes in our hospital from January 2012 to January 2016 were followed up with an average of 3.8 years. The incidence of cardio-cerebrovascular diseases between diabetes combined with hyperlipidemia group (195 patients) and diabetes group (200 patients) were made a comparison. Multivariable Cox’s proportional hazards regression model was used to analyze the effect of hyperlipidemia on the incidence of cardio-cerebrovascular diseases in patients with type 2 diabetes.

**Results:**

Diastolic blood pressure, systolic blood pressure, high-density lipoprotein, low-density lipoprotein, body mass index and hyper-sensitive C-reactive protein were higher in diabetes combined with hyperlipidemia group than in diabetes group (*P* < 0.05). At the end of the follow-up period, all-cause mortality, cardio-cerebrovascular diseases mortality, and the incidence of myocardial infarction, cerebral infarction, cerebral hemorrhage and total cardiovascular events were significantly higher in diabetes combined with hyperlipidemia group than in diabetes group (*P* < 0.05). The analysis results of multivariable Cox’s proportional hazards regression model showed that the risks of myocardial infarction and total cardiovascular events in diabetes combined with hyperlipidemia group were respectively 1.54 times (95%*CI* 1.13–2.07) and 1.68 times (95%*CI* 1.23–2.24) higher than those in diabetes group. Population attributable risk percent of all-cause mortality and total cardiovascular events in patients with type 2 diabetes combined with hyperlipidemia was 9.6% and 26.8%, respectively.

**Conclusions:**

Hyperlipidemia may promote vascular endothelial injury, increasing the risk of cardio-cerebrovascular diseases in patients with type 2 diabetes. Medical staffs should pay attention to the control of blood lipids in patients with type 2 diabetes to delay the occurrence of cardio-cerebrovascular diseases.

## Background

The latest data from the International Diabetes Federation revealed that there were 387 million diabetics worldwide in 2014. In high-income countries, type 2 diabetes accounted for 85%~ 95% of diabetes, which might be higher in middle-income and low-income countries. By the year 2035 the number of diabetic patients is expected to increase by 55% to 600 million. The burden of diabetes is growing more severe as a result of the increasing number of deaths from diabetes and medical costs [1]. Diabetic patients often combined with metabolic disorders like hypertension, and hyperlipidemia, easily lead to cardio-cerebrovascular diseases like coronary heart disease, which is a risk factor leading to death [2]. Diabetes complications, cardio-cerebrovascular diseases are the common factors that cause the death of patients. According to statistics, more than 75% of diabetic patients die from cardio-cerebrovascular diseases every year [3]. According to statistics, there are some 30%–40% of diabetic patients in China with hyperlipidemia [4]. Hyperlipidemia and diabetes are independent risk factors of cardio-cerebrovascular diseases [5, 6], and the coexistence of the two can increase the risk of cardio-cerebrovascular diseases [7, 8]. An epidemiological survey showed the incidence of acute stroke in patients with high hyper-sensitive C-reactive protein (hs-CRP) level was two times higher than healthy people and myocardial infarction was three times higher [9]. We followed up 395 patients with type 2 diabetes in our hospital from January 2012 to January 2016, and analyzed as follow.

## Methods

### Clinical data

Three hundred ninety five patients with type 2 diabetes in our hospital from January 2012 to January 2016 were divided into diabetes combined with hyperlipidemia group (195 patients) and diabetes group (200 patients). Whether patients have a family history of diabetes were inquired. Inclusion criteria: ① fasting plasma glucose (FPG) ≥7.0 mmol/L; ② FPG < 7.0 mmol/L but patients were previously diagnosed with diabetes and were using hypoglycemic agent [10]. Exclusion criteria: ① previous history of myocardial infarction; ② previous history of stroke; ③ refusing to sign an informed consent; ④ incomplete baseline data.

### Diagnostic criteria

Diabetes diagnosis based on the guidelines for the prevention of diabetes in China in 2010 and hypertension diagnosis based on the guidelines for the prevention of hypertension in China in 2011 [11]: systolic blood pressure (SBP) ≥ 140 mmHg and/or diastolic blood pressure (DBP) ≥ 90 mmHg (1 mmHg = 0.133 kPa), or normal blood pressure but taking anti-hypertensive drugs. Definition of obesity [11]: body mass index (BMI) ≥ 28 kg/m^2^. Definition of hyperlipidemia [12]: total cholesterol ≥ 5.72 mmol/L. Definition of smoking history: one or more cigarettes every day, lasting over 1 year. Definition and diagnostic criteria of cardiovascular disease: cardio-cerebrovascular events include fatal and nonfatal cardio-cerebrovascular events. Cardiac events include acute myocardial infarction and sudden cardiac death. Acute myocardial infarction is diagnosed according to the diagnostic criteria developed by the Chinese Medical Association’s Cardiovascular Disease Branch [13]. Sudden cardiac death is diagnosed according to the diagnostic criteria of the American College of Cardiology/American Heart Association/European Society of Cardiology Committee in 2006 [14]. Cerebrovascular events including cerebral infarction and cerebral hemorrhage, are diagnosed according to the diagnostic criteria of Cerebrovascular Disease Classification (1995) developed by the Fourth National Conference on Cerebrovascular Disease [15]. Cardiovascular events include heart failure, myocardial infarction, and sudden death. The total cardio-cerebrovascular events include myocardial infarction, cerebral infarction and cerebral hemorrhage. When the total cardio-cerebrovascular events are counted, one event occurring two or more times is recorded only 1 time, ending with the time and event of the first endpoint event.

### Follow-up method

Follow-up period arranged from January 2012 to January 2016. New-onset cardio-cerebrovascular events were collected every three months. First, professional clinicians collected the main cardio-cerebrovascular events records of participants through the Zhengzhou City Medical Insurance Management Center. Subsequently, cardio-cerebrovascular physicians consulted patients’ medical records to confirm the occurrence time and type of cardio-cerebrovascular events and analyzed the change of hs-CRP for patients during the follow up.

### Statistical analysis

SPSS13.0 software was used to analyze the data. Measurement data were showed as mean ± standard deviation ($\overline{x}$*±s*). Comparison between groups was made by t test. Enumeration data were analyzed by Chi-square test. There was a significant difference at *P* < 0.05. The person-time morbidity and mortality in diabetes group and diabetes combined with hyperlipidemia group were calculated respectively. The differences in the incidence of cardio-cerebrovascular events between the two groups were compared. Multivariable Cox’s proportional hazards regression model was used to analyze the factors affecting cardio-cerebrovascular events and to calculate hazard ratio (HR) in each group. Population attributable risk percent (PAR%) was calculated according to the formula [16], and it was used to analyze the effect of hypertension on cardio-cerebrovascular events in patients with diabetes.

$$ \mathrm{PAR}\%=\mathrm{Morbidity}\ \left(\mathrm{HR}\hbox{-} 1\right)/\left[\mathrm{Morbidity}\ \left(\mathrm{HR}\hbox{-} 1\right)+1\right]\times 100\% $$

## Result

### Baseline data

Three hundred ninety five patients with type 2 diabetes in our hospital were followed up from January 2012 to January 2016, with an average follow-up period of 3.8 years. There were 195 patients in diabetes combined with hyperlipidemia group and 200 patients in diabetes group. There were 28 patients below 45 years old, 102 patients of 45~ 54 years old, 158 patients of 55~ 64 years old, 77 patients of 65~ 74 years old, and 36 patients over 75 years old, with an average of 58.9 (29~ 88) years old. Age, SBP, DBP, low-density lipoprotein (LDL), high-density lipoprotein (HDL), BMI, total cholesterol, smoking ratio and hs-CRP all were higher in diabetes combined with hyperlipidemia group than in diabetes group, however, FPG was lower (*P* < 0.05) (Table 1).

**Table 1:** Baseline data ($\overline{x}$±s)

Group	Patients	Age (years)	DBP (mmHg)	SBP (mmHg)	BMI (kg/m^2^)
Diabetes	200	52 ± 0.25	75 ± 13	120 ± 14.5	24.8 ± 0.5
Diabetes combined with hyperlipidemia	195	58 ± 0.18^a^	98 ± 9^a^	149 ± 13.5^a^	26.9 ± 0.8^a^
Group	Total cholesterol (mmol/L)	HDL (mmol/L)		LDL (mmol/L)	Smoking (%)
Diabetes	5.2 ± 0.89	1.75 ± 0.18		1.36 ± 0.12	23.8%
Diabetes combined with hyperlipidemia	5.9 ± 0.67^a^	2.68 ± 0.38^a^		2.45 ± 0.36^a^	38.5%^a^
Group	FPG (mmol/L)	Hypertension (%)	Obesity (%)	Follow-up period (year)	hs-CRP (mg/L)
Diabetes	9.45 ± 0.16	21.8	19.8	4.0 (3.8–4.2)	1.86 ± 0.45
Diabetes combined with hyperlipidemia	9.04 ± 0.38^a^	35.9^a^	29.7^a^	4.2 (3.7–4.4)^a^	9.65 ± 0.82^a^

Note: *DBP* diastolic blood pressure, *SBP* systolic blood pressure, *BMI* body mass index, *HDL* high-density lipoprotein, *LDL* low-density lipoprotein, *FPG* fasting plasma glucose, *hs-CRP* hyper-sensitive C-reactive protein

^a^*P* < 0.05

### All-cause mortality and cardio-cerebrovascular events mortality

All-cause mortality and cardio-cerebrovascular events mortality in diabetes combined with hyperlipidemia group were higher than those in diabetes group during the follow-up. Patients below 65 years old in diabetes combined with hyperlipidemia group had higher all-cause mortality and cardio-cerebrovascular events mortality than those in diabetes group (*P* < 0.05). There was no difference in all-cause mortality and cardio-cerebrovascular events mortality of patients over 65 years old between the two groups. All-cause mortality and cardio-cerebrovascular events mortality of males and females in diabetes combined with hyperlipidemia group were all higher than those in diabetes group (*P* < 0.05). All-cause mortality and cardio-cerebrovascular events mortality of patients with family history of diabetes in diabetes combined with hyperlipidemia group were all higher than those in diabetes group (*P* < 0.05), and for patients without family history of diabetes there was no difference between the two groups (Table 2).

**Table 2:** All-cause mortality and cardio-cerebrovascular events mortality (/1000 persons/year)

Patients	Group	All-cause death	Mortality (95%CI)	Cardio-cerebrovascular events death	Mortality (95%CI)
Total	Diabetes	18	4.5 (3.67–5.98)	5	1.3 (0.98–1.65)
	Diabetes combined with hyperlipidemia	32	8.4 (6.09–9.24)^a^	14	3.7 (2.97–4.02)^a^
< 65 years	Diabetes	9	2.2 (1.87–2.48)	3	0.6 (0.38–0.78)
	Diabetes combined with hyperlipidemia	21	5.4 (4.81–5.94)^a^	8	1.7 (1.42–2.02)^a^
≥ 65 years	Diabetes	26	5.2 (4.89–5.42)	5	0.9 (0.88–1.02)
	Diabetes combined with hyperlipidemia	23	5.4 (5.11–5.64)	6	1.1 (0.97–1.12)
Female	Diabetes	5	1.1 (0.87–1.28)	2	0.4 (0.28–0.53)
	Diabetes combined with hyperlipidemia	17	4.4 (4.22–4.74)^a^	7	1.5 (1.42–1.62)^a^
Male	Diabetes	11	3.1 (2.87–3.28)	4	0.7 (0.64–0.83)
	Diabetes combined with hyperlipidemia	25	4.9 (4.82–5.14)^a^	9	1.9 (1.83–2.02)^a^
Family history	Diabetes	10	2.8 (2.57–2.98)	6	0.6 (0.58–0.73)
	Diabetes combined with hyperlipidemia	25	5.9 (5.53–6.14)^a^	12	1.9 (1.83–2.02)^a^
No family history	Diabetes	17	3.6 (3.37–3.88)	10	1.6 (1.48–1.73)
	Diabetes combined with hyperlipidemia	20	3.9 (3.53–4.14)	12	1.9 (1.83–2.02)

*CI* confidence interval

Note: ^a^*P* < 0.05, which is compared with the same patients in diabetes group

### Incidence of cardio-cerebrovascular events

Fifty seven of 395 patients suffered from cardio-cerebrovascular events. There were 31 patients with myocardial infarction, 18 patients with cerebral infarction and 8 patients with cerebral hemorrhage. The incidence of myocardial infarction, cerebral infarction, cerebral hemorrhage and total cardio-cerebrovascular events in diabetes combined with hyperlipidemia group were all higher than those in diabetes group (*P* < 0.05). Patients below 65 years old in diabetes combined with hyperlipidemia group had higher incidence of myocardial infarction and total cardio-cerebrovascular events than those in diabetes group (*P* < 0.05). The incidence of myocardial infarction and total cardio-cerebrovascular events of females in diabetes combined with hyperlipidemia group were higher than those in diabetes group. The incidence of myocardial infarction, cerebral infarction, cerebral hemorrhage and total cardio-cerebrovascular events of males in diabetes combined with hyperlipidemia group were higher than those in diabetes group (*P* < 0.05) (Table 3).

**Table 3:** Incidence of cardio-cerebrovascular events (/1000 persons/year) (95%CI)

Patients	Group	Total cardio-cerebrovascular events	Myocardial infarction	Cerebral infarction	Cerebral hemorrhage
Total	Diabetes	3.5 (3.37–3.78)	2.3 (2.17–2.88)	1.5 (1.37–1.78)	0.8 (0.66–0.98)
	Diabetes combined with hyperlipidemia	6.4 (6.09–7.04)^a^	4.4 (4.11–4.91)^a^	3.4 (3.24–3.81)^a^	1.6 (1.42–1.81)^a^
< 65 years	Diabetes	3.6 (3.57–4.18)	2.5 (2.37–2.78)	1.8 (1.68–1.96)	0.7 (0.6–0.78)
	Diabetes combined with hyperlipidemia	5.2 (5.01–5.5)^a^	4.1 (3.8–4.21)^a^	1.7 (1.64–1.81)	0.6 (0.45–0.81)
≥ 65 years	Diabetes	2.6 (2.47–2.88)	1.8 (1.67–1.94)	1.2 (1.08–1.36)	0.6 (0.57–0.78)
	Diabetes combined with hyperlipidemia	2.8 (2.71–2.91)	1.7 (1.66–2.01)	1.3 (1.24–1.41)	0.5 (0.45–0.65)
Females	Diabetes	2.5 (2.37–2.75)	1.9 (1.78–2.04)	1.1 (1.01–1.26)	0.5 (0.38–0.61)
	Diabetes combined with hyperlipidemia	4.1 (4.01–4.2)^a^	3.1 (2.8–3.21)^a^	1.2 (1.04–1.31)	0.6 (0.55–0.71)
Males	Diabetes	3.2 (3.13–3.4)	2.5 (2.31–2.65)	1.7 (1.57–1.88)	0.9 (0.76–1.08)
	Diabetes combined with hyperlipidemia	6.1 (5.89–6.44)^a^	4.8 (4.61–5.2)^a^	2.8 (2.74–3.01)^a^	1.4 (1.22–1.61)^a^

*CI* confidence interval

Note: ^a^*P* < 0.05, which is compared with the same patients in diabetes group

### Multivariable analysis of the effect of hyperlipidemia on cardio-cerebrovascular events in patients with type 2 diabetes

**Fig. 1:**
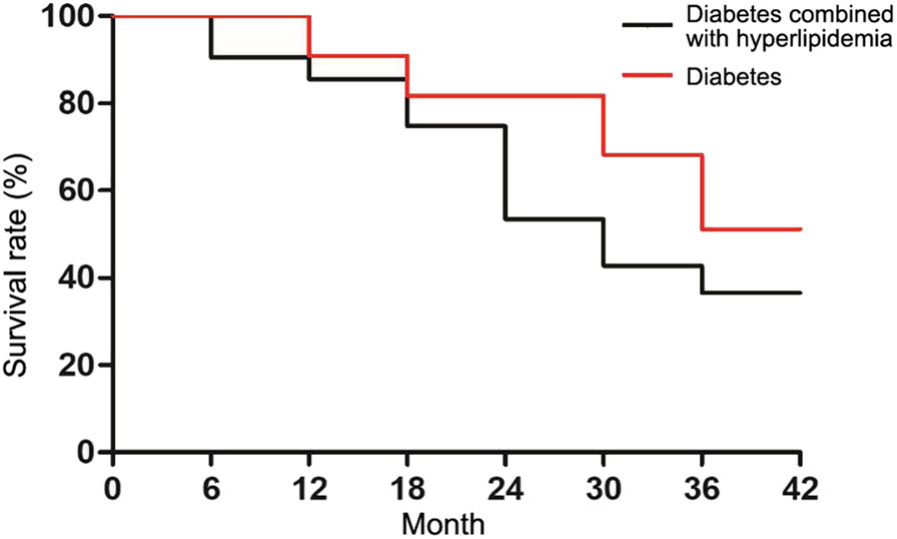
Survival rate in diabetes group and diabetes combined with hyperlipidemia group at the end of the follow-up

In this research, the incidence of hyperlipidemia was 44.6%, and the incidence of diabetes combined with hyperlipidemia was 49.4%. Among of them the incidence of hyperlipidemia in patients with all-cause death, cardio-cerebrovascular events death, myocardial infarction, cerebral infarction, cerebral hemorrhage and total cardio-cerebrovascular events were 67.5%, 73.2%, 69.8%, 70.4%, 65.8% and 61.3%, respectively. All-cause death, cardio-cerebrovascular events death, myocardial infarction, cerebral infarction, cerebral hemorrhage and total cardio-cerebrovascular events were used as the dependent variables. Hypertension, age, gender, FPG, smoking, obesity, high cholesterol, low density lipoprotein cholesterol (LDL-C) and high density lipoprotein cholesterol (HDL-C) were used as the independent variables. The differences in age, gender, obesity, hypertension and smoking were corrected. Multivariable Cox’s proportional hazards regression model was used to analyze these factors. The results showed that after covariant correction the risk of myocardial infarction and total cardio-cerebrovascular events in diabetes combined with hyperlipidemia group were 1.54 times (95%*CI* (confidence interval) 1.13–2.07) and 1.68 times (95%*CI* 1.23–2.24) higher than those in diabetes group (*P* < 0.05). PAR% of hyperlipidemia on all-cause death and total cardio-cerebrovascular events in patients with type 2 diabetes were 9.6% and 26.8% (Table 4). At the end of the follow-up statistics revealed that the survival rate in diabetes group was significantly higher than that in diabetes combined with hyperlipidemia group (Fig. 1).

**Table 4:** Multivariable analysis of the effect of hyperlipidemia on cardio-cerebrovascular events in patients with type 2 diabetes

Events	HR (95%CI)	*P*	Hyperlipidemia morbidity (%)	PAR (%)
All-cause death	1.21 (0.89–1.31)	> 0.05	67.5	9.6
Cardio-cerebrovascular death	1.33 (0.97–1.42)	> 0.05	73.2	20.2
Myocardial infarction	1.41 (1.12–1.56)	< 0.05	69.8	22.1
Cerebral infarction	1.32 (1.09–1.47)	> 0.05	70.4	25.7
Cerebral hemorrhage	0.98 (0.78–1.12)	> 0.05	65.8	28.3
Total cardio-cerebrovascular events	1.78 (1.65–1.98)	< 0.05	61.3	26.8

*PAR%* Population attributable risk percent, *CI* confidence interval

Note: Correction factors are age, gender, obesity, hypertension and smoking

## Discussion

Diabetes is a disease caused by multi-source etiology including heredity, social factors, life-style and environment [17]. The incidence of diabetes is believed to be related to age, family history of diabetes, obesity levels and types, and insulin resistance. However, recent studies have found that cardio-cerebrovascular disease is a major risk factor for the safety of patients with type 2 diabetes, especially for the safety of the elderly. In addition to heredity factor, the pathogenesis of cardio-cerebrovascular disease is more related to the lifestyle and dietary pattern of patients. It is reported that about 75% of diabetes patients die from cardio-cerebrovascular disease every year [18]. The study found that due to biological regulation dysfunction of insulin, diabetes patients commonly accompanied by lipid metabolism disorder and were complicated by hyperlipidemia [19]. Diabetes combined with hyperlipidemia could accelerate the progress of atherosclerosis and increase the incidence of cardiovascular disease [20]. Hyperlipidemia combined with abnormal hs-CRP may also be a key factor in promoting vascular endothelial injury of patients with hypertension and the incidence of cardiovascular disease. There is growing evidence that low level of CRP is closely related to the risk factor of cardiovascular disease, such as hypertension and hyperlipidemia, and that the elevation of CRP level can increase the incidence of heart disease and stroke for patients with hypertension. And therefore CRP is a proinflammatory factor related to the occurrence and development of atherosclerosis. During the formation of atherosclerotic plaque CRP, complement complexes and foam cells deposited on arterial wall. CRP can be combined with lipoprotein to activate complement system, producing a large number of inflammatory mediators, releasing oxygen free radicals, causing endangium injury, vasospasm and unstable plaque rupture, aggravating the luminal stenosis caused by atherosclerosis and promoting the occurrence of myocardial infarction [21]. In our research, DBP, SBP, HDL, LDL, BMI and hs-CRP in diabetes combined with hyperlipidemia group were all higher than those in diabetes group. All-cause mortality, cardio-cerebrovascular diseases mortality, and the incidence of myocardial infarction, cerebral infarction, cerebral hemorrhage and total cardiovascular events were significantly higher in diabetes combined with hyperlipidemia group than in diabetes group (*P* < 0.05). The results of multivariable Cox’s proportional hazards regression model showed that the risk of myocardial infarction and total cardio-cerebrovascular events in diabetes combined with hyperlipidemia group were 1.54 times (95%*CI* 1.13–2.07) and 1.68 times (95%*CI* 1.23–2.24) higher than those in diabetes group. This result might be caused by lipid metabolism disorder. Lipid metabolism disorder, as a common complication of type 2 diabetes, easily caused angiosclerosis, inducing cardio-cerebrovascular disease like coronary heart disease and cerebral infarction. Over-high LDL-C level in blood could lead to the accumulation of LDL-C in the coronary artery, which prompted the formation of atheromatous plaque to obstruct the lumen, causing ischemia and hypoxia of myocardium [22, 23]. Hyperlipidemia could damage the vascular endothelial cells, increasing the permeability of vascular wall. Thus the plasma lipoprotein penetrating the inner membrane induced the elimination of macrophages, the proliferation of the smooth muscle cells, atherosclerosis, even prompting the formation of atheromatous plaque and angiostenosis. Therefore, the reduction of blood lipid levels played an effective prevention function on cardio-cerebrovascular disease [24]. The study also proved that lipid metabolism disorder was positively correlated with the incidence of ischemic cardio-vascular disease [25]. Hypercholesterolemia is one of the most important risk factors for atherosclerotic cardiovascular disease (coronary heart disease and ischemic stroke), and coronary heart disease is one of the leading causes of death in diabetics [26, 27]. Some studies have revealed that the risk of hypertension and diabetes is growing at a different rate, and that the risk of hypertension and diabetes is increased after 25 months and 27 months of follow-up. Hypertension is a common risk factor of cardiovascular disease, and rational treatment and scientific management can reduce its incidence [28–30]. Some studies found that the increased risk of coronary artery disease in diabetics might be partly attributable to diabetes-related lipoprotein abnormalities. Several II level prevention trials including diabetic patients, have demonstrated the effectiveness of lower LDL-C in preventing coronary artery disease deaths. In patients with type 2 diabetes, although the blood lipids value improved, lipid metabolism disorder persisted even if the blood glucose was controlled in the optimal range [31–33]. In this research, all-cause mortality and cardio-cerebrovascular events mortality of patients with family history of diabetes in diabetes combined with hyperlipidemia group were all higher than those in diabetes group, and for patients without family history of diabetes there was no difference between the two groups. The results of family history of diabetes showed the risk of cardiovascular disease increased even though these patients did not have symptoms of early diabetes or diabetes. The symptoms of early diabetes were the further manifestation of early atherosclerosis. The synergistic effect of systemic inflammation and high concentration of blood sugar could damage the function of vascular endothelial cells and induce atherosclerosis lesion. Therefore the effective blood lipid control in early diabetes or in patients with family history of diabetes could reduce the incidence of cardiovascular disease [34].

## Conclusions

Hyperlipidemia increases the risk of cardio-cerebrovascular disease in patients with type 2 diabetes, and hyperlipidemia combined with the elevation of hs-CRP may induce higher risk of cardio-cerebrovascular disease in patients with type 2 diabetes. Medical staffs should take preventive measures according to individual situation to reduce or delay the risk and the onset time of cardiovascular disease in patients with diabetes.

## Abbreviations

BMI: Body mass index
CI: Confidence interval
DBP: Diastolic blood pressure
FPG: Fasting plasma glucose
HDL: High-density lipoprotein
HDL-C: High density lipoprotein cholesterol
HR: Hazard ratio
hs-CRP: hyper-sensitive C-reactive protein
LDL: Low-density lipoprotein
LDL-C: Low density lipoprotein cholesterol
PAR%: Population attributable risk percent
SBP: Systolic blood pressure
